# Intrinsic Brain Activity in Temporal Lobe Epilepsy With and Without Depression: Insights From EEG Microstates

**DOI:** 10.3389/fneur.2021.753113

**Published:** 2022-01-04

**Authors:** Yueqian Sun, Guoping Ren, Jiechuan Ren, Qun Wang

**Affiliations:** ^1^Department of Neurology, Beijing Tiantan Hospital, Capital Medical University, Beijing, China; ^2^National Center for Clinical Medicine of Neurological Diseases, Beijing, China; ^3^Collaborative Innovation Center for Brain Disorders, Beijing Institute of Brain Disorders, Capital Medical University, Beijing, China

**Keywords:** temporal lobe epilepsy, depression, microstates parameter, EEG, resting state

## Abstract

**Background:** Depression is the most common psychiatric comorbidity of temporal lobe epilepsy (TLE). In the recent years, studies have focused on the common pathogenesis of TLE and depression. However, few of the studies focused on the dynamic characteristics of TLE with depression. We tested the hypotheses that there exist abnormalities in microstates in patients with TLE with depression.

**Methods:** Participants were classified into patients with TLE with depression (PDS) (*n* = 19) and patients with TLE without depression (nPDS) (*n* = 19) based upon the Diagnostic and Statistical Manual of Mental Disorders, Fifth Edition (DSM-V). Microstate analysis was applied based on 256-channel electroencephalography (EEG) to detect the dynamic changes in whole brain. The coverage (proportion of time spent in each state), frequency of occurrence, and duration (average time of each state) were calculated.

**Results:** Patients with PDS showed a shorter mean microstate duration with higher mean occurrence per second compared to patients with nPDS. There was no difference between the two groups in the coverage of microstate A–D.

**Conclusion:** This is the first study to present the temporal fluctuations of EEG topography in comorbid depression in TLE using EEG microstate analysis. The temporal characteristics of the four canonical EEG microstates were significantly altered in patients with TLE suffer from comorbid depression.

## Introduction

Epilepsy is a chronic neurological disorder characterized by recurrent episodes of spontaneous seizures, affecting nearly 1–2% population of the world (1). Epileptic seizures are caused by the imbalance of excitatory and inhibitory neurotransmitters in central nervous system, thus leading to abnormal synchronous firing occurs in the involved neural networks of brain (2). The most prevalent type of focal epilepsy is temporal lobe epilepsy (TLE). In this population, comorbidity burden is high and psychiatric comorbidities are frequently encountered (3), such as depression, which is the most common psychiatric comorbidity. The recently reported prevalence numbers of depression comorbidity in patients with TLE vary from 30 to 50% (4, 5). Comorbid depression has been further linked not only to high rates of suicide and decreased life expectancy, but it is also a greater risk factor for developing refractory epilepsy (5, 6).

However, the precise mechanism of comorbid depression in TLE is not yet fully elucidated. In the recent years, studies have focused on the neurobiological basis of TLE and depression, suggesting that a common pathogenesis may exist. The pathogenesis includes disorders of the endocrine system (7–10), abnormal neurotransmitter balance (11, 12), changes in immune-related biochemical indicators (13, 14), abnormal glucose metabolism (15, 16), inflammation (17), and neurogenesis (15). Spenser et al. (18) highlighted that epilepsy and depression have similar networks with postulated roles in neuropsychiatric disorders that overlap, providing a theoretical basis for the high prevalence of comorbid depression disorders in epileptic patients. Therefore, knowledge of the physiological mechanisms at an intrinsic network level is essential to patients with TLE with depression.

Most recent studies have indicated that brain neural activity changes dynamically through time and, thus, provides abundant information of neural characteristics for epilepsy and depression (19, 20). The electroencephalography (EEG) activity is segmented into limited amounts of scalp electrical topographies of certain time periods (60–120 ms) duration and then dynamically changing into a different state that remains stable again (8, 21). Each successive signal is referred to as “microstate” and transitions between microstates are thought to reflect coordinated interactions among large-scale distributed brain networks (22). In the resting state, only four specific topographies (termed microstates A, B, C, and D) are able to explain most of the global variance of EEG signals (>65%) (22). Microstate metrics included the duration (average time of each state remains stable), occurrence (the number of times it occurred per second), coverage (the percentage of total time spent in each state), and microstate syntax (transition probabilities from each microstate class to another) (23). Simultaneous EEG-functional MRI (fMRI) has reported association of the microstates A and B with phonological and visual and microstates C and D with salience and attention networks (24). Most of the studies conducted focused on fMRI and few on EEG (22). With the emergence of dense array EEG technologies, the recording of more accurate electrical source imaging has become available. Due to its advantages of submillisecond temporal, high-spatial resolution, and high signal-noise ratio, high-density EEG covering all the relevant neural regions has become more likely to reveal underlying mechanisms.

The purpose of this study was to examining deviant resting-state EEG microstate dynamics in patients with TLE with depression as compared to patients with TLE without depression. We hypothesized that in patients with TLE suffer from comorbid depression, the temporal characteristics of the four canonical microstate maps will be significantly altered. In this study, we applied 256-channel high-density EEG to reveal the microstate dynamic changes in patients with TLE with depression over time.

## Materials and Methods

### Study Design and Participants

This retrospective study was based on data of patient collected from Beijing Tiantan Hospital from January 2019 to June 2021. The diagnosis was conducted by at least two well-trained neurologists. Inclusion criteria were: (i) diagnosed as TLE according to the criteria established by the International League Against Epilepsy (25); (ii) age over 18 years old; (iii) the epileptogenic zone was localized to the temporal lobe by continuous video EEG evaluation; and (iv) being seizure free for at least 72 h. Exclusion criteria were: (i) previous neurosurgery; (ii) cognitive impairment assessed using the Mini-Mental State Examination; (iii) a history of other neurological disorders, except epilepsy; and (iv) use of antidepressant medications prior to this study. Depression was ascertained using the Fifth Edition of Diagnostic and Statistical Manual of Mental Disorders (DSM-V) criteria and depressive severity was assessed using the 24-item version of the Hamilton Depression Rating Scale (HAMD24). A total of 38 patients with TLE were enrolled and divided into patients with depression (PDS, *n* = 19) and patients without depression (nPDS, *n* = 19). This study was approved by the Hospital Ethics Committee and all the patients signed informed consent forms.

### Electroencephalography Acquisition and Preprocessing

Prior to EEG measurements, patients were requested to lie comfortably in the supine position and relax their facial muscles. During the acquisition, subjects remained awake with their eyes closed to reduce artifact signals due to eye movements and avoid deliberate mental activities. Data were recorded using 256-channel high-density EEG recordings (EGI System 400; Electrical Geodesic Incorporation, Oregon, USA, band pass filter: 0.1–70 Hz, sampling rate: 1,000 Hz, and impedance <30 kΩ with a recording reference at the vertex). We subjected EEG to rule out the presence of interictal EEG discharges for all the patients. For further analysis, the number of electrodes was reduced from 256 to 203 channels in order to minimize artifacts from facial or neck muscles.

Each EEG dataset was segmented into 2 s non-overlapping epochs and bad channels were removed with subsequent interpolation. If a channel was bad for 20% or more of the epochs, the channel was flagged as bad for all the epochs; if more than 15% of the channels in a single segment were labeled as bad, the whole segment was rejected. Electroencephalography epochs contaminated by movement artifacts were manually discarded from subsequent analysis. Independent component analysis was employed to remove components associated with persistent ocular and electrical artifacts. At the end, an artifact-free data were selected per subject from which estimating the parameters for the microstate analyses.

### Microstate Analysis

**Figure 1:**
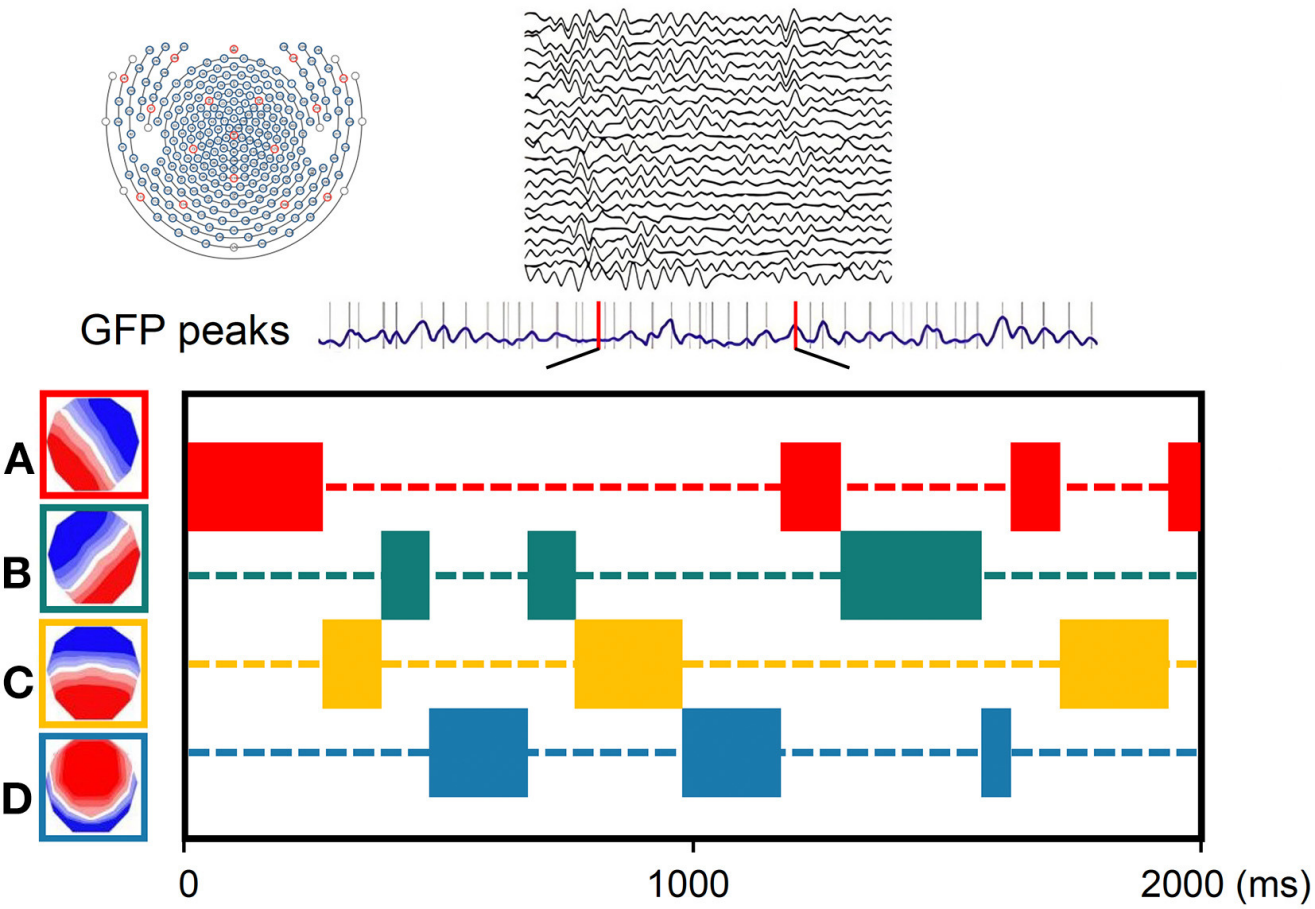
Schematic of the method of microstate analysis. The global field power (GFP) (drawn in purple) is calculated at each instant of the 256-channel electroencephalography (EEG) recording. Original maps at the times of maximal GFP are plotted and assigned into the four group model maps labeled A, B, C, or D.

The atomize-agglomerate hierarchical cluster (AAHC), a modified k-means to provide unique clusters for microstate analysis, was used to generate clusters of EEG topographies (26). Electroencephalography was bandpass filtered (0.2–20 Hz) (27) and average rereferenced. The polarity of the topographical maps was disregarded (26, 28). The global field power (GFP) (spatial standard deviation as a function of time) is subsequently calculated across EEG channels as a function of time to quantify synchronous activity from all of the electrodes at every timepoint (29). Global field power peaks have been previously proven to represent moments of highest signal-to-noise ratios and strongest field potentials. The topographic maps are always steady during the high GFP, and immediately after, change to the next topographic map, once GFP reaches a minimum peak (30). In microstate analysis, the topographies of GFP peaks are regarded to be discrete microstates, whereas dynamic changes in EEG signals as variations of these states (23). Cluster analysis was conducted first at the individual template maps level and then at group levels. To facilitate comparisons with previous studies, we categorized the microstate maps into four categories (A–D) on the basis of previous study (22) (Figure 1). Spatial correlations between each map at group level and the topographies (maps) at the GFP peaks of the original EEG signals at individual level were calculated. Therefore, microstate maps were used to determine the backward fitting to the original map topography at each GFP peak according to maximum spatial correlation. The timepoints between two GFP peaks were obtained using nearest-neighbor interpolation. For each microstate map, four temporal parameters including duration, occurrence, coverage, and microstate syntax were calculated.

### Statistical Analysis

Statistics were calculated with the SPSS Statistics version 25 (IBM Corporation, Armonk, New York USA). For the differences in microstate duration, occurrence, and coverage, data were analyzed by using the Wilcoxon rank-sum test. For the differences in microstate syntax, the non-random transition probabilities from each microstate to another were counted; these numbers were normalized to fractions of all between-class transitions of the subjects. Given four classes, we, thus, obtained for each subject 12 values for all the possible sequence doublets. False discovery rate (FDR) was used for multiple testing correction (FDR *q*-values < 0.01). Besides, comparisons between groups were conducted with the two-tailed *t*-tests. *p*-values < 0.05 were considered as statistically significant.

## Results

### Demographics and Clinical Variables

Demographics and pertinent clinical data are given in Table 1. There were no significant differences in age, gender, years of education, course of epilepsy, and localization of TLE between patients with and without depression. The HAMD scores differed significantly between the two groups [21.0 (15.0, 35.0) vs. 6.0 (2.0, 6.0), *p* < 0.01]. As described in the methods, patients were categorized into the PDS group (HAMD score ≥ 7) and the nPDS group (HAMD score < 7).

**Table 1:** Demographic and clinical characteristics of participants.

**Variable**	**PDS**	**nPDS**	***P*-value**
	**(*n* = 19)**	**(*n* = 19)**	
Age (year)	26.0 (23.0, 30.0)	29.0 (19.0, 31.0)	0.58
Sex			0.71
Male	13 (68%)	15 (79%)	
Female	6 (32%)	4 (21%)	
HAMD score	21.0 (15.0, 35.0)	5.0 (2.0, 6.0)	<0.01
Epilepsy duration (year)	10.0 (6.0, 15.0)	7.0 (4.0, 17.0)	0.41
Education years	12.0 (9.0, 13.0)	9.0 (8.0, 12.0)	0.46
Lateralization			0.75
Left	9 (47%)	11 (58%)	
Right	10 (53%)	8 (42%)	

*PDS, patients with temporal lobe epilepsy with depression; nPDS, patients with temporal lobe epilepsy without depression; HAMD, 24-item Hamilton Depression Rating Scale*.

### Electroencephalography Microstate Analysis

**Figure 2:**
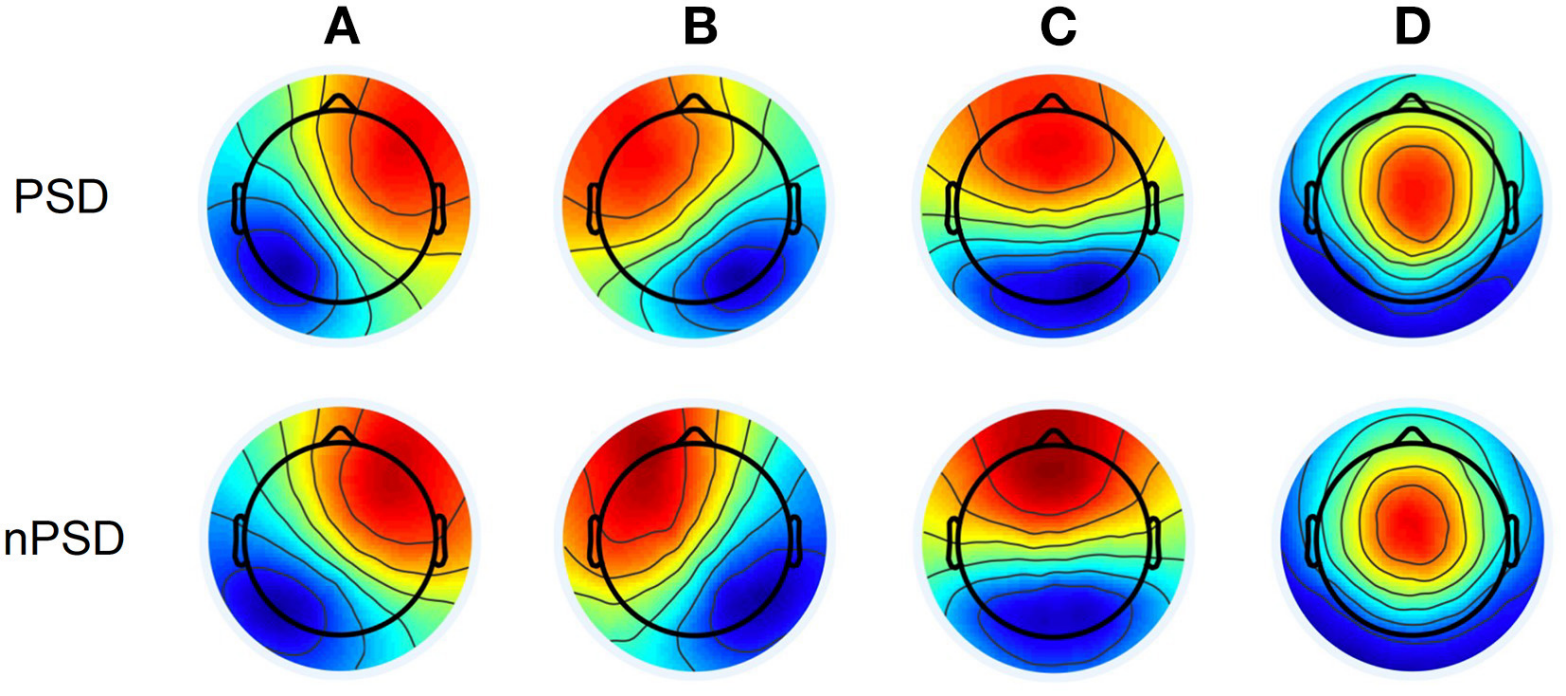
Microstate topographic maps. PDS, patients with temporal lobe epilepsy with depression; nPDS, patients with temporal lobe epilepsy without depression.

Group dominant microstate maps are shown in Figure 2. They were highly similar to the categories observed in previous studies (22). The orientation of microstate A is from right frontocentral to left occipital-parietal; the orientation of microstate B is from left frontocentral to right parieto-occipital; the orientation of microstate C is from prefrontal to occipital; the orientation of microstate D is from frontocentral to occipital. The four microstate classes are labeled accordingly and explained 77.0% (SD: 3.9%) of the total variance across PDS and 78.2% (SD: 3.1%) across nPDS, respectively. These results suggested no statistical difference between these two groups (*p* = 0.45).

**Figure 3:**
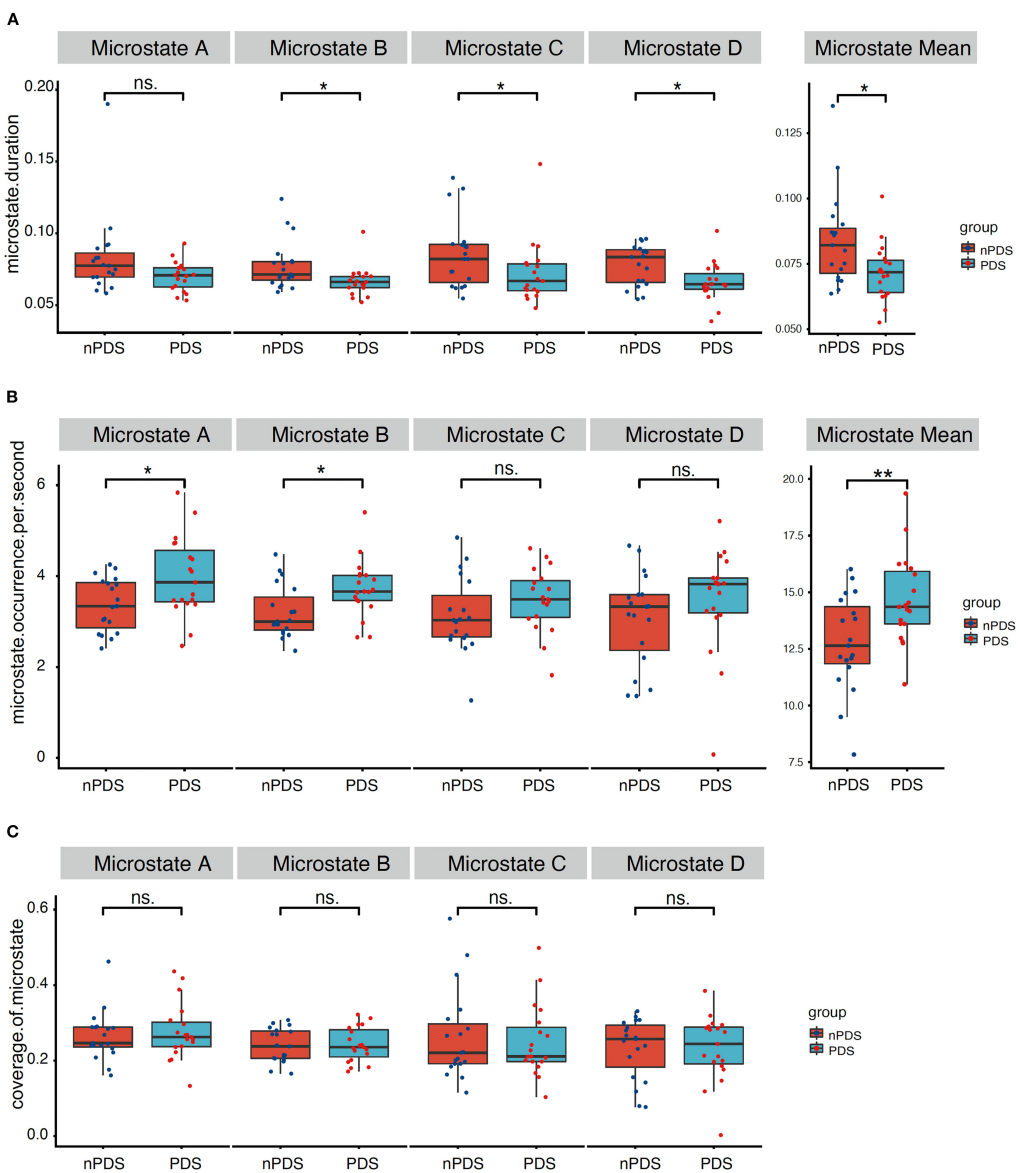
Temporal characteristics of microstate. Comparison between the two groups of microstate duration, occurrence per second, and coverage for each microstate class separately. **(A)** correspond to the duration of microstate map A–D and mean duration, **(B)** correspond to the occurrence per second of microstate map A–D and mean occurrence, **(C)** correspond to the coverage of microstate map A–D. *p*-values result from the Wilcoxon rank-sum test between two groups. PDS, patients with temporal lobe epilepsy with depression; nPDS, patients with temporal lobe epilepsy without depression. *p < 0.05, **p < 0.01.

**Figure 4:**
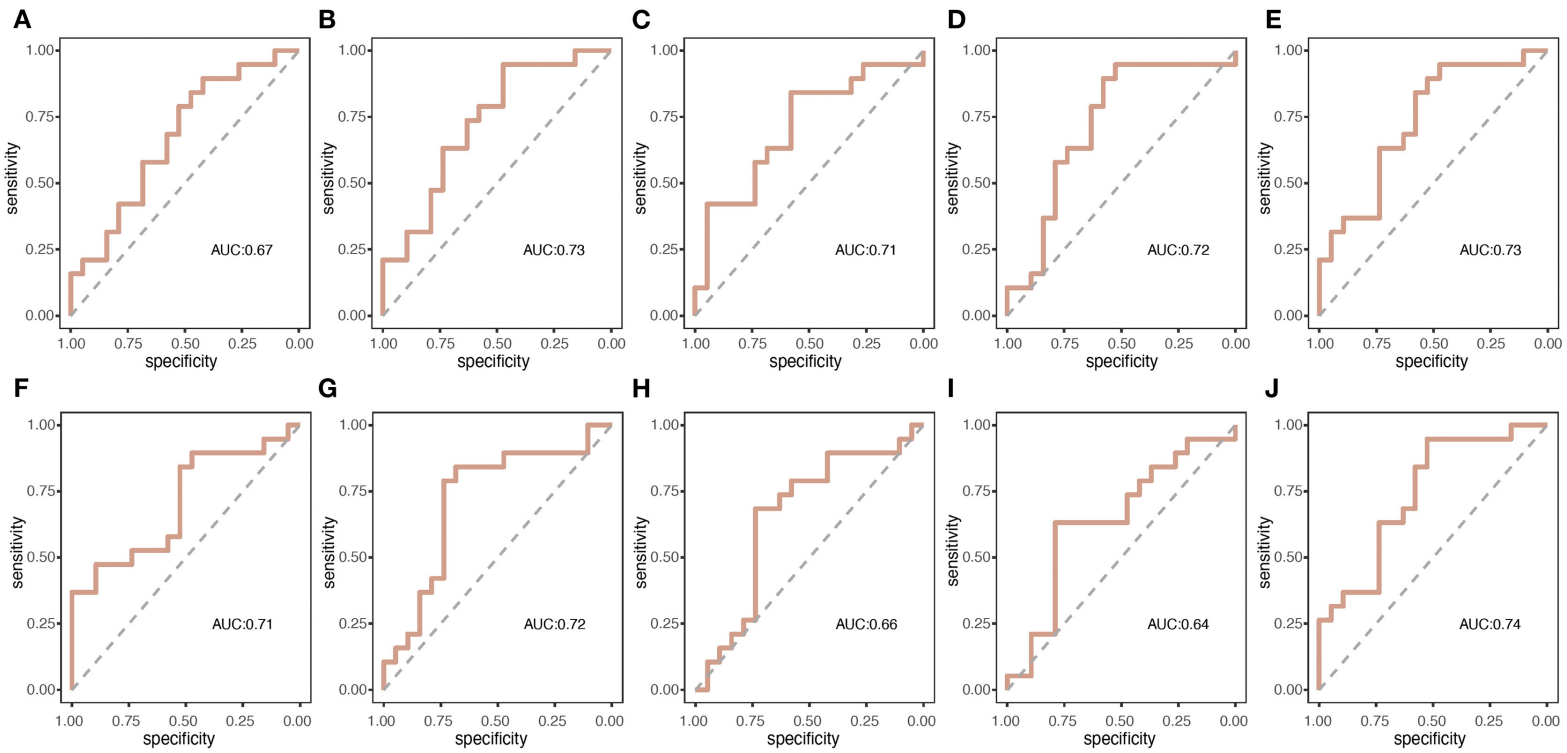
The summary receiver operating characteristic (ROC) curves for the two groups of microstate duration and occurrence per second. **(A–E)** correspond to the duration of microstate map A–D and mean duration, respectively; **(F–J)** correspond to the occurrence per second of microstate map A–D and mean occurrence, respectively.

Figure 3 presents the duration, occurrence, and coverage for the four microstate classes. Microstate mean duration ranged from 38.8 to 190.1 ms for the different microstate classes. Patients with nPDS had longer duration on average than patients with PDS (*p* = 0.013) (Table 2; Figure 3). Microstate occurrence ranged between 0.07 and 5.84. Compared with patients with nPDS, patients with PDS displayed a less mean occurrence per second (*p* = 0.010). Proportion of total time covered by the different microstates varied from 0.3 to 57.6%. There was no difference between the two groups in the coverage of microstates A–D. In microstate A, compared with nPDS, the microstate occurrence of patients with PDS increased. But, there was no difference between the two groups with duration and coverage (Table 4). In microstate B, a higher occurrence per second with a shorter duration was found in patients with PDS compared with nPDS. The coverage did not reveal statistical significance between groups. In microstates C and D, the duration was shorter than in nPDS. No other temporal characteristics differed between the two groups (Figure 1; Tables 2–4). In this study, we showed the receiver operating characteristic (ROC) curves for microstate duration and occurrence (Figure 4). There was an adequate discrimination for patients with PDS and nPDS: on ROC analysis, all the areas under the ROC curves of both duration and occurrence shown in the figure were larger than 0.6 and were 0.73 for mean duration and 0.74 for mean occurrence.

**Table 2:** Duration of microstates A to D and mean of two groups.

	**PDS (*n* = 19)**	**nPDS (*n* = 19)**	***P*-value**
A	0.071 (0.062, 0.076)	0.078 (0.069, 0.089)	0.075
B	0.066 (0.062, 0.066)	0.071 (0.066, 0.807)	0.017
C	0.067 (0.060, 0.079)	0.082 (0.063, 0.925)	0.027
D	0.065 (0.060, 0.075)	0.083 (0.065, 0.089)	0.018
mean	0.072 (0.064, 0.077)	0.082 (0.070, 0.090)	0.013

*PDS, patients with temporal lobe epilepsy with depression; nPDS, patients with temporal lobe epilepsy without depression*.

**Table 3:** Occurrence per second of microstates A–D and mean of two groups.

	**PDS (*n* = 19)**	**nPDS (*n* = 19)**	***P*-value**
A	3.862 (3.418, 4,644)	3.337 (2.738, 3.879)	0.030
B	3.661 (3.453, 4.018)	2.999 (2.779, 3.714)	0.020
C	3.489 (3.066, 3.945)	3.030 (2.642, 3.882)	0.096
D	3.824 (3.148, 3.959)	3.327 (2.205, 3.595)	0.140
Mean	14.353 (13.604, 16.050)	12.644 (11.696, 14.649)	0.010

*PDS, patients with temporal lobe epilepsy with depression; nPDS, patients with temporal lobe epilepsy without depression*.

**Table 4:** Contribution of microstates A to D and results from comparison between two groups.

	**PDS (*n* = 19)**	**nPDS (*n* = 19)**	***P*-value**
A	0.262 (0.236, 0.307)	0.247 (0.232, 0.289)	0.73
B	0.236 (0.202, 0.287)	0.238 (0.206, 0.280)	0.82
C	0.211 (0.197, 0.301)	0.221 (0.192, 0.310)	0.98
D	0.244 (0.186, 0.290)	0.257 (0.156, 0.300)	0.93

*PDS, patients with temporal lobe epilepsy with depression; nPDS, patients with temporal lobe epilepsy without depression*.

Between any microstate measures above and the severity of depression according to the HAMD score, there was no significant correlation (*p* > 0.05).

## Discussion

We found a marked increase in the dynamic changes of the brain network in patients with PDS compared with patients with nPDS. Collectively, patients with PDS had shorter mean durations and higher occurrences than patients with nPDS. This is the first study to present the temporal fluctuations of EEG topography in patients with TLE with depression using EEG microstate analysis, which robustly affirmed alterations in a specific subset of subsecond functional states of brain.

To the best of our knowledge, only a handful of studies focused on spontaneous EEG microstates in TLE or depression (31–36). However, no studies are currently available that address the altered large-scale network dynamics in TLE with depression using microstate analysis. Compared to patients with nPDS, patients with PDS showed a decreased overall resting-state microstate duration of microstates B, C, and D and an increase of occurrence of microstate A and B.

Previous resting-state fMRI studies demonstrated that microstate class A was highly correlated with the auditory network (24). Involved regions included bilateral superior and middle temporal gyri, which is relevant to voice processing. Besides recognition of the brain involvement, the source of topography of EEG microstate has attracted much attention (37). Left lateral activity in temporal lobe, insula, medial prefrontal cortex, and the occipital gyri has been proposed as a major sources of microstate A (37, 38). Temporal lobe epilepsy and depression share common involved brain regions including the temporal, frontal lobes, amygdala, hippocampus, entorhinal cortex, subcortical structures including basal ganglia and thalamus, and the connecting pathways (39). The findings of fMRI studies showed the functional changes in the superior temporal gyrus in patients with a major depression (40, 41). A number of studies in the auditory domain document alterations in auditory system in major depressive disorder (MDD) (42–44). Higher occurrence of the microstate A has been shown to be related to greater depression severity in MDD. This conclusion was corroborated by our results, which showed the significant alterations of microstate A in patients with TLE with depressive symptom.

This study showed decreased duration and increased occurrence of class B in patients with PDS. Microstate B could mirror the alterations in resting-state visual networks (24). Major depressive disorder had been reported to show abnormal functional connectivity (FC) within visual regions (45, 46). Abnormal visual and auditory networks have increasingly been recognized as a core feature of depression (47), which could explain, at least in part, by the fact that there were differences of microstates A and B between patients with PDS and nPDS.

The alterations in microstate C were not found to be unique to epilepsy. Prior microstate studies have reported increased frequency not only in epilepsy (31), but also in schizophrenia (48, 49) and syndrome of 22q11 deletion (50). Our results indicated that microstate map C could reflect the combined effect of comorbidity depression that patients living with TLE might harbor. Besides, precuneus activity often is implicated in microstate C (38) and it has been perceived in patients with TLE with MDD that the spontaneous brain activity is altered in precuneus (51). Therefore, we inferred that the alteration of microstate C in the nPDS group is likely driven by brain network involved precuneus. In addition, microstate map C is predominated by a task inhibitory alpha level (52). Intriguingly, past study has shown that depressive patients had decreased alpha (53, 54), which might be the key factor that causes vigilance in depressive behavior. Furthermore, a phenomenon that frontal EEG alpha asymmetry has been described in depressed patients (55, 56). All of the above could support the suggestion that in this study, the PDS group showed alterations of microstate C compared to the nPDS group.

Microstate D was found to be negatively related to BOLD signal changes in right-lateralized dorsal and ventral areas of the frontal-parietal networks involved in attentional reorienting and switching (22, 57). The default mode network (DMN) is disrupted in patients with TLE with MDD according to previous studies. Moreover, increased activation in the DMN including midline thalamus, precuneus, hippocampus, ventral anterior cingulate cortex, and prefrontal cortex was found in patients with TLE with depression (58), implying that midline structure is one of the key brain structures involved in the emotional modulation and hyperactivation in these regions disrupt normal emotional function. Consistent with these results, we found that microstate D showed shorter duration in patients with TLE with depression.

In addition, we found a lower duration of B to D microstates in patients with PDS. Duration is the key parameter in microstate analysis because accurate timing is of great importance to manage the flow of information the brain has to deal with at each moment to exert their functionality (59). This speaks to the increased dynamical changes of the brain network structures in patients with TLE with depressive symptom.

There was no correlation of the HAMD scores with microstate parameters. Such an observation may result from limitations to the utilization of the HAMD scale. As far as we know, most of the previous studies on depression used other depression scales such as Beck Depression Inventory-II (33) or Montgomery–Åsberg Depression Rating Scale (32) to assess correlation between depressive severity and microstate parameters. Therefore, another depression scale may have to be employed in future studies to capture this correlation.

## Limitations

Several study limitations need to be acknowledged. First, the sample size may not be sufficient. Therefore, the study needs replications with larger sample sizes. Second, this study considers only temporal and not spatial dynamics or time-frequency analysis. Third, more detailed and comprehensive scales are required to assess the severity of depression. Furthermore, given that none of our patients with PDS received antidepressant therapy as EEG was recorded, we cannot come to any further conclusions with respect to the potential effect of antidepressants on the microstate parameters of patients with PDS.

## Conclusion

We analyzed the altered resting-state EEG microstate dynamics measured with high-density EEG in TLE with comorbid depression and compared them to those without comorbid depression. Classic microstate analysis provide insight into subsecond time scale whole-brain dynamics in depression comorbidity in epilepsy. Large scale EEG microstate network alterations cast a perspective on the neuronal networks underlying depression in TLE. The high spatiotemporal resolution of high-density EEG provides a detailed understanding of functional network.

## Data Availability Statement

The raw data supporting the conclusions of this article will be made available by the authors, without undue reservation.

## Ethics Statement

The studies involving human participants were reviewed and approved by Beijing Tiantan Hospital Ethics Committee. The patients/participants provided their written informed consent to participate in this study.

## Author Contributions

QW concepted, designed, and supervised the study. YS acquired the data, analyzed and interpreted the data, provided statistical analysis, had full access to all of the data in the study, responsible for the integrity of the data and the accuracy of the data analysis, and drafted the manuscript. GR, JR, and QW critically revised the manuscript for important intellectual content. All authors read and approved the final version of the manuscript.

## Funding

The study was financially supported by the National Key R&D Program of China grant (2017YFC1307500), the Capital Health Research and Development of Special grants (2016-1-2011 and 2020-1-2013), the Beijing-Tianjin-Hebei Cooperative Basic Research Program (H2018206435), the Beijing Natural Science Foundation (Z200024), and the National Natural Science Foundation of China (81801280 and 81601126).

## Conflict of Interest

The authors declare that the research was conducted in the absence of any commercial or financial relationships that could be construed as a potential conflict of interest.

## Publisher's Note

All claims expressed in this article are solely those of the authors and do not necessarily represent those of their affiliated organizations, or those of the publisher, the editors and the reviewers. Any product that may be evaluated in this article, or claim that may be made by its manufacturer, is not guaranteed or endorsed by the publisher.
